# Expertise in performance assessment: assessors’ perspectives

**DOI:** 10.1007/s10459-012-9392-x

**Published:** 2012-07-31

**Authors:** Christoph Berendonk, Renée E. Stalmeijer, Lambert W. T. Schuwirth

**Affiliations:** 1 Institute of Medical Education, Faculty of Medicine, University of Berne, Konsumstrasse 13, 3010 Berne, Switzerland; 2 Faculty of Health, Medicine and Life Sciences, Department for Educational Development and Research, Maastricht University, Maastricht, The Netherlands; 3 Flinders Innovation in Clinical Education, Health Professions Education School, Flinders University, Adelaide, Australia

**Keywords:** Assessment, Assessment for learning, Decision making, Expertise development, Performance appraisal

## Abstract

The recent rise of interest among the medical education community in individual faculty making subjective judgments about medical trainee performance appears to be directly related to the introduction of notions of integrated competency-based education and assessment for learning. Although it is known that assessor expertise plays an important role in performance assessment, the roles played by different factors remain to be unraveled. We therefore conducted an exploratory study with the aim of building a preliminary model to gain a better understanding of assessor expertise. Using a grounded theory approach, we conducted seventeen semi-structured interviews with individual faculty members who differed in professional background and assessment experience. The interviews focused on participants’ perceptions of how they arrived at judgments about student performance. The analysis resulted in three categories and three recurring themes within these categories: the categories *assessor characteristics*, *assessors’ perceptions of the assessment tasks*, and the *assessment context*, and the themes *perceived challenges*, *coping strategies*, and *personal development*. Central to understanding the key processes in performance assessment appear to be the dynamic interrelatedness of the different factors and the developmental nature of the processes. The results are supported by literature from the field of expertise development and in line with findings from social cognition research. The conceptual framework has implications for faculty development and the design of programs of assessment.

## Introduction

Around the world, undergraduate and graduate medical curricula are being reformed. Where in the past the main focus was on well-defined learning outcomes, today the notion of integrated competencies is rapidly gaining ground (ten Cate and Scheele 2007), posing new challenges to assessment. Firmly rooted in the psychometric discourse, the traditional objective of assessment was to ascertain objectively whether students had attained the desired results (Hodges 2006). This type of assessment is characterized by highly standardized settings where the influence of individual assessors was minimized.

With the introduction of integrated competency-based education, however, several drawbacks of the pursuit of objective assessment emerged (Norman et al. 1991). First, the traditional psychometric notion of assessment OF learning does not fit well with the philosophies underlying competency-based education which emphasize the notion of assessment FOR learning from the constructivist discourse (Shepard 2000; Krupat and Dienstag 2009). Second, the psychometric approach is considered to be too reductionist (Huddle and Heudebert 2007) for the assessment of higher order competencies, such as the ability to work in a team, professional behavior, and self-reflection, which are increasingly deemed to be essential for medical professionals but cannot be meaningfully assessed detached from the authentic context (Kuper et al. 2007). The assessment of integrated competencies inevitably has to rely on individual judgments of student performance in the real learning and working environment. The use of subjective judgment in assessment is supported not only by theoretical notions regarding assessment but also by empirical research indicating that subjective judgments are in fact widely employed in performance assessment in the health professional context (Ginsburg et al. 2010). Although subjectivity does not necessarily imply unreliability (Van der Vleuten et al. 1991; Swanson et al. 1995), it is nevertheless of critical importance to reduce the risk of arbitrary judgment, especially when assessment relies on single judgments by single assessors. Before incorporating such assessments in a program of assessment, measures should be in place to optimize the credibility and defensibility of the assessments, which in turn should be grounded in a good understanding of human judgment in performance assessment.

In the literature on human judgment, three main fields of research stand out: *bias and heuristics, natural decision making*, and *social cognition* theory. Research on *biases and heuristics* investigates factors that make human judgment so notoriously fallible. Studies comparing human probability judgment to actuarial judgment invariably show the superiority of the latter type of judgment and the fallibility of the former (Dawes et al. 1989), while Kahneman and Tversky highlight numerous sources of error and bias that contribute to suboptimal human decision making (Tversky and Kahneman 1974).

Research into *naturalistic decision making* focuses on how humans are able to arrive at satisfactory decisions, especially in situations where actuarial methods are infeasible, such as ambiguous, ill-defined, uncertain situations where often quick decisions have to be made (Klein 2008). Central to these theories is the assumption that human judgment in such situations relies on quickly matching the problem to exemplars in long-term memory to identify not *the* *best* solution but a *good enough* solution (Simon 1956). *Social cognition* research acknowledges that the interpersonal and social environment inevitably impacts on individual human decision makers. In other words, decisions depend not only on the actual problem at hand but also on the motivations and personal goals of individual decision makers and the local practices wherein the decision making takes place (Levy and Williams 2004).

These perspectives, however, do not shed sufficient light on the processes involved in subjective assessment of student performance to inform faculty development activities that can enhance the quality of such assessments. Insofar as medical education research has addressed this topic, the focus has been on the reporting of substandard performance by assessors (Dudek et al. 2005; Cleland et al. 2008), with studies showing that assessors’ decisions whether or not to report substandard performance can only be understood in terms of ‘motivated social judgment’. Noel and colleagues analyzed the accuracy of experienced clinicians’ observations of medical trainees performance (Noel et al. 1992) and concluded that examiners often ignore or overlook the available information, a finding that seems to resonate with findings from research on *biases and heuristics* in human judgment. Important steps in building a knowledge base about performance assessment have been made by Govaerts et al. They revealed striking resemblances between the cognitive structure of diagnostic (Schmidt and Rikers 2007) and assessor expertise (Govaerts et al. 2011).

With increasing expertise, assessors typically became more efficient in obtaining a good representation of performance and provide richer and more interpretative descriptions of trainee performance. However, while increased assessment expertise resulted in quicker and less effortful processing of information in complex examination situations, there was no similar effect found for prototypical situations (Govaerts et al. 2012), suggesting that assessor expertise depends at least partly on an expanding repertoire of scripts, a phenomenon that is also reported for expertise development in other areas (Boreham 1994). Nevertheless, the factors that contribute to the development of expertise in assessment and their interactions still remain largely uncharted territory. We conducted a qualitative study to explore these factors with the aim of building a preliminary model that can enhance our understanding of assessor expertise.

## Methods

As the aim of our study was to explore factors that contribute to assessor expertise, we conducted a qualitative study using a grounded theory approach (Glaser and Strauss 1967).

### Study context

The study was conducted in the setting of the ‘physician-clinical investigator’ program, a four-year graduate entry program of Maastricht University, the Netherlands, leading to a master’s degree in medicine and with a special focus on the translation of results of basic research to patient care and of patient problems to basic science research. The program consists of modules in a spiral curriculum that emphasizes collaborative learning. Student performance is assessed with a variety of methods, such as essays, scientific reports, case presentations, oral examinations, assessment of (professional) behavior during small group work, OSCEs, and student-patient encounters. According to the assessment guidelines at the time of our study, each assessment served a formative as well as a summative purpose. All the teachers have to attend a mandatory two-day faculty development workshop on the skills required for the different teaching and assessment roles in the program. We conducted our study in this particular program because of the numerous instances where teachers assess student performance. All the teachers who participated in the study also taught in the regular six-year undergraduate curriculum, and since the assessment approaches of the two programs are comparable, we invited the teachers to also talk about their assessment experiences outside the ‘physician-clinical investigator’ program whenever they considered this relevant.

### Research team

The research team consisted of one MD with 4 years of experience in educational development, an educationalist with 7 years of experience in educational research and development with a special focus on qualitative research, and an MD who is a professor of medical education with 20 years of experience in medical education research.

### Participants and ethical procedure

Participants were purposively sampled with the help of key informants, and had to meet the selection criteria of being involved in the ‘physician-clinical investigator’ program and having experience in assessing student performance of clinical or non-clinical tasks. All participants received information about the project several days before their individual interviews and were asked to sign and return a written informed consent form, explaining the goals of the research and the expected outcomes. They received no remuneration. Information disclosed by the participants (transcript) was only discussed within the research team where strict confidentiality was maintained. The twenty educators who were invited to participate in the study represented a broad range of gender, experience in assessment, clinical and basic science, the humanities, and biomedical science. At the time of the planning of the study and the collection of the data there was no relevant institutional review board for medical education studies and the university’s institutional review board ruled that this type of research was exempt from ethical approval.

### Semi-structured interviews

In view of the sensitivity of the topic and the mutual dependency of the participants as colleagues in a small-scale program, we decided to conduct individual interviews. A tentative interview guide was developed based on the research question and informed by literature in the domains of medical education, judgment and decision making, and expertise. After one pilot interview to test the questions, some refinements were made. The pilot interview was not included in the analysis. The research team discussed the resulting interview guide, which consisted of the following topics: information used in forming a judgment, reference criteria, (different) ways of arriving at a judgment and the time needed to do so, student factors influencing decision making, abilities deemed essential for assessors, perceived pressure, and conflicting views on performance assessment. The guide was used as the starting point for the interview but any promising information provided by the interviewees was pursued as well. All interviews lasted approximately 60–90 min and were conducted by the same investigator (CB) between April and June 2010. All interviews were conducted in English, which was the only common but foreign language for the interviewer as well as for all the interviewees.

### Analysis

All the interview recordings were transcribed verbatim and entered into qualitative data analysis software (Atlas-ti 6.2). The principal investigator (CB) concurrently analyzed and collected the data to ensure that the interviews were effectively eliciting the types of description that were anticipated, to allow for the exploration of interesting side themes, and to estimate the point of saturation (Kennedy and Lingard 2006). Saturation was reached after seventeen interviews. Based on the initial analysis the research team agreed on a preliminary coding scheme. Codes were established in an iterative process in which the coding informed subsequent discussions within the research team and the discussions in turn informed the coding process. A second researcher (LS) repeated the coding to enhance the credibility of the analysis. Next, connections between the codes were explored and categories identified. Through constant comparison of codes and categories, the data were aggregated into themes. Throughout this process the researchers evaluated literature that was considered pertinent to the emerging theory. The data was presented in a format that was used by Westerman and colleagues to highlight the interrelatedness of different codes (Westerman et al. 2010). Although Westerman’s research addressed a different research question, their table was helpful in clarifying the higher order themes that were identified. The research team discussed the results until consensus was reached.

## Results

Of the seventeen teachers that we interviewed, seven were female and ten male. Eight interviewees held an MD degree (seven clinicians and one epidemiologist), six had a background in basic science and three a background in psychology or sociology. Five participants conducted assessments in the clinical as well as in the academic setting. The mean age was 50.5 years (range 36–62 years) and the mean number of years of experience with assessment was 16.6 years (range 3–33 years).

The analysis resulted in three categories (*assessors characteristics*, *assessors’ perceptions of the assessment tasks*, and the *assessment context*) and three recurring themes within these categories (*perceived challenges*, *coping strategies*, and *personal development*). For the sake of comprehensiveness we first present the three categories before describing their interactions. Table 1 shows a schematic overview of the main findings for the categories and the interrelated themes.

**Table 1:** Categories and themes within the emergent conceptual framework

Themes	Categories		
	Assessor characteristics	Assessors’ perceptions of the assessment tasks	Assessment context
Perceived challenges	Level of knowledge about Content itself What students know Self-efficacy in own assessment abilities	Varying beliefs about Purpose Authenticity Guidance Lack of tangible standard	Perceived external pressure Desire to protect the ‘teacher role’
Coping strategies	Get involved with assessment Practice with peers, formal training	Adapting rules Adapting standard Normative standard	Adapting standard Second opinion
Personal development	Feeling of insecurity diminishes Expertise develops	Ownership of the assessment task ‘Absolute’ standard	Adapting examination rules and curriculum

The category of *assessor characteristics* concerns participants’ perceptions of their own knowledge and self-efficacy and how these impact on assessment. The *task* category contains statements about *what*, *why* and *how* assessors are instructed to assess and participants’ perceptions of these guidelines as well as remarks about the standards they adopted in judging student performance. The *context* category relates to aspects inherent in the setting in which the assessment is conducted and to participants’ perceptions of how these contextual factors influence their decision making.

### Assessor characteristics

At some point during the interview, almost every participant mentioned feelings of insecurity in relation to assessment. A fair number of aspects that were said to give rise to uncertainty could be attributed to characteristics of the assessors. Participants regarded *content knowledge* as a prerequisite for credible and fair assessment of student performance, and regretted that they were quite frequently put in a position where this requirement was not met. Participants who had witnessed non-content experts assessing students in their own field of professional expertise seriously questioned the fairness and credibility of such assessments.

“Sometimes they invite or they appoint some people and they say you have to do these evaluations but they haven’t got a clue. And I think that is quite unfair, because if you are a dietician and you are supposed to do a mental status, well, I felt more or less insulted. Because I have had, you know, it is part of my profession. You are asking a dietician who has never seen a psychiatric patient to do an evaluation; that is not fair. That is not fair to me and not fair to the student.” [Interviewee 15]

The participants did not believe that a lack of content knowledge could be easily remedied by short interventions, such as a 30-min training session, or by providing detailed checklists.

“…because even just checking ‘done’ or ‘not done’, it is difficult …you also have to check sometimes on what level it has been done: good, bad or intermediate.” [Interviewee 1]

Content knowledge was considered necessary but not sufficient to perform an assessment task well. *Knowledge about the level of knowledge* that students were expected to show at a particular stage in the curriculum was also considered to be critical. A lack of that type of knowledge was identified as a probable cause of uncertainty.

“Well, what should they know and what’s not relevant at this stage of their study? That’s what we’re still struggling with all day …is this something they should know and is this something they shouldn’t know? No idea.” [Interviewee 10]

Finally, the degree of s*elf*-*efficacy* with respect to the assessment task influenced whether assessors felt uncertain or confident in making assessment decisions.

“Who am I to judge these students? Who am I to tell you it’s okay or it’s not okay?” [Interviewee 10]

Participants mentioned three main strategies that helped them handle the challenges of assessment. The first strategy consisted in being selective in accepting assessment tasks. A number of participants stated that they avoided certain assessment tasks they “didn’t like” and preferred tasks where they felt they could make a meaningful contribution. Gaining experience by doing, however, was the most common strategy. It is through practical experience that assessors come to understand what they can expect from students. However, many participants felt that experience alone did not suffice to achieve assessment expertise, and some participants explicitly indicated that they viewed assessment as a set of skills that could and had to be learned. Collaboration was another strategy, involving for instance attendance of a formal teach the teacher/assessor program, but hands-on practical experience followed by peer feedback was seen as the most powerful collaborative learning experience for assessors.

“Just before I came here, I was discussing with a colleague of mine who was checking the questions first and then she came back to me: Did I do this right? Because she also has to learn. So I was helping her by being again helped by somebody who is older than me. So we try to teach each other this way.” [Interviewee 5]

With increasing experience feelings of insecurity subsided and decision making became less fraught with doubt and anxiety.

When asked how they formed an opinion about student performance, experienced participants often used terms like *“gut feeling”* and *“you just see it”*. They seemed to chunk bits of information into meaningful ‘gestalt’- like patterns. A typical answer of an experienced assessor to the question “what were the reasons why you judged this student as outstanding?” was:

“She asked the questions which were necessary to be asked. I had a good feeling in what she did.” [Interviewee 6]

Only after probing questions from the interviewer did this teacher give more details about important aspects in assessing student performance during patient encounters.

“I look how their way of asking questions is. How do they interact with the patients? How do they get out the information that they want? How much room in the anamnesis [history taking] are they giving the patients; is it long questions, open questions, short questions? Are they really listening well to the patients? …I try to get a complete picture of the student.” [Interviewee 6]

The closer the assessment task was to the interviewee’s own field of expertise and the more experienced the interviewee was in assessment, the richer these task specific performance descriptions became.

### Assessors’ perceptions of the assessment tasks

A second set of aspects that was considered problematic and a source of uncertainty related to the tension between participants’ individual *beliefs* about assessment and the school’s examination rules and regulations. For instance, there could be differences of opinion as to *why* an assessment was (should be) done, in other words about the *purpose of the assessment*. Participants also perceived tension between their roles as teacher and assessor. Many participants “wanted to help the students” and viewed assessment primarily as a stimulus for learning, whereas many assessment forms emphasize grading and pass/fail decisions. Interestingly, the double role of teacher and assessor was not perceived exclusively as a source of tension. Quite the opposite in fact, with teachers arguing that integrating these roles was of vital importance for them to be able to identify with the whole process and create meaning.

“Teaching, of course, is assessing. And assessing is teaching. Then it is an interesting role. Because then you give meaning to your feedback.” [Interviewee 16]

Discrepancies between participants’ opinions and the official guidelines related not only to the purpose of assessment but also to *how* a certain task was best assessed, especially if it entailed more elusive or ill-defined concepts like professional behavior and communication. A major concern of the participants was the lack of *authenticity* of these assessments.

“We had a special item … the behavior towards the patient. But I mean, it is so short [interaction time between student and simulated patient] that I doubt if it works … it is not real.” [Interviewee 14]

There was also concern about the forms used to document assessments. Participants expressed contradictory desires for both guidance and freedom. Most assessors would welcome some kind of grid that could serve as a “backbone” for judgments, but the standard forms rarely met their needs.

When the official rules ran counter to their beliefs about assessment, participants resorted to two main strategies: non-compliance with the rules, if they felt an assessment served no useful purpose;

“Well to be honest, I do not think I really did a good assessment there. Apart from filling in some kind of paper I remember, some kind of thing that I needed to fill in, I think I even asked them [the students] to fill it in themselves and have it signed by me, something like that. So that was an easy job and maybe not according to the rules, but it worked.” [Interviewee 2]

and applying the rules rather loosely, if they gave learning priority over assessment.

“And maybe it is not, actually it might not be sufficient at the end [of the module] … [but] if I see improvement, then it is often enough for me, because there is ample time for them to further improve.” [Interviewee 9]

When participants’ views and the assessment regulations with regard to a specific task were irreconcilable participants sometimes decided to subject the whole assessment process to careful scrutiny, sharing with peers ideas and beliefs about how to make the assessment task more meaningful. In cases where this resulted in modification of the task, teachers often experienced a strong sense of ownership of the assessment program.

Besides diverging beliefs about assessment, the *lack of a clear standard* for judging observed performance was considered problematic. Identifying outstanding or very weak performance was usually quite straightforward, but decisions about performance “in the grey area” (at the pass/fail boundary) posed a much greater challenge. Assessors coped with this by comparing the performances of different students on the same task.

“‘Oh well, that first one [student] was good’, but you only realize that after you’ve seen five or six [students]. So then you change the overall score, a little higher or a little lower, whatever is needed. So there’s always a comparison.” [Interviewee 10]

As their experience with a particular assessment task grew, participants came to depend less on comparisons between students and seemed to develop something like an *absolute* standard, which could be quite idiosyncratic, implicit, and geared to a specific task they performed repeatedly at a specific point in the curriculum.

“Now I have taken these exams for a few years now, I think, I know what is pass and fail.” [Interviewee 3]

### Assessment context

Assessments of student performance on authentic tasks are enacted within a social context, and participants considered aspects of this context, especially potentially adverse consequences to themselves, before communicating their judgments. Differences between assessors’ ‘private’ and ‘public’ judgments could be caused by the teacher’s wish to protect the educational relationship with a student or by pressure from external sources, such as student appeals, which could militate against fail decisions in the absence of airtight evidence.

“There’s a big problem with exam rules and all the possibilities to fight results where the students usually get their way and really very irritating if you have to work hard to uphold protests from students.” [Interviewee 7]

A common strategy for dealing with these perceived pressures was to *lower the standards*.

“Especially if students don’t meet your expectations, then you end up into problems and then you have to probably narrow down the personal learning goals.” [Interviewee 4]

Another strategy that was used especially in cases of *external pressure* was to ask a peer assessor for a *second opinion*.

Assessors with a strong commitment to education might serve on committees that set the examination rules or develop the curriculum, finding themselves in a position where they can influence *what* is assessed and *how* assessment is conducted.

### Dynamic interactions between assessor, task, and context

The framework describes *assessor characteristics, task perception,* and *context* as three separate entities, but the analysis revealed strong interrelationships and mutual influences between the factors. For instance, beliefs about *how* assessment should be conducted were not a fixed trait of individual assessors, and perceptions regarding the purpose of assessments might vary depending on the content and the place in the curriculum of a particular assessment task. One of the assessors, who emphasized the learning aspect of assessment of professional behavior in the first year of the curriculum, saying

“I think it’s very good to give feedback to each other, to say positive and negative things. … I think that’s a very good part because indeed you can already learn in an early phase how you behave.” [Interviewee 17]

adopted a strictly summative approach to an oral examination at the end of the curriculum.

“It’s their last examination, so they are a doctor, a month later. I’m not going to help them, they just need to know it.” [Interviewee 17]

Performance assessment appeared to be characterized not only by the impact of interrelated factors but also by its developmental nature. Assessment tasks that are perceived as difficult by novice assessors become less challenging with increasing experience. However, due to the task specificity of assessment this development is not a one-way street. For instance, when a new assessment format is introduced, even hugely experienced assessors will have to overcome some initial uncertainty while getting used to the new format. In summary, interrelated factors and developmental dynamics appeared to hold the key to a better understanding of the processes involved in performance assessment.

## Discussion

We qualitatively explored factors that contribute to assessor expertise to obtain insights that we could use as building bricks for a preliminary model of assessor expertise. We identified three interacting categories (*assessor characteristics*, *assessors’ perceptions of the assessment tasks*, and *assessment context*) and three interrelated developmental themes (*perceived challenges,* *coping strategies,* and *personal development)*.

Our findings suggest that expertise is pivotal to performance assessment, and the model we propose does indeed resonate with central notions from the literature on expertise development: domain specific knowledge, longitudinal, hands-on, practical experience, and the need for feedback from credible sources. The centrality of domain-specific knowledge in expertise was established several decades ago (Chi et al. 1985), and seems particularly relevant in situations where ill-defined problems with multiple solutions force decision makers to choose between alternatives (Simon 1973). In assessing the performance of a medical student running a busy outpatient clinic for example, an assessor has to decide whether to focus on the ability to work efficiently or the ability to engage in empathic student-patient relationships. Also, when assessors with little domain-specific knowledge have difficulty processing divergent pieces of information in judging student performance on ill-structured problems, they are likely to resort to non-domain specific heuristics to guide their judgment, unlike domain experts, who have developed ways to structure such problems in their area of expertise (Voss and Post 1985). For example, in assessing a student taking a patient history an assessor who lacks domain specific knowledge may rely on generic heuristics and judge performance elements such as starting the interview with an open question and ending with a summary of the patient’s problems, whereas an expert assessor is more likely to focus on whether the student establishes a good rapport with the patient and has a sound grasp of the problem at hand.

Our framework also emphasizes that with increasing practice assessors develop ‘performance scripts’ which facilitate assessment. Experienced assessors appear to be sensitive to cues that correlate, albeit not necessarily causally, with (future) student performance. This process is akin to the development of clinical expertise, where medical trainees acquire ‘clinically relevant information about the enabling conditions of disease’ which they incorporate into ‘illness scripts’, a process that is largely based on practical experience (Schmidt and Rikers 2007).

Our findings are also consistent with the pivotal role of feedback in the literature on expertise development (Ericsson 1993). In performance assessment, feedback not only seems to improve (assessment) performance but also fosters a shared vision of performance standards among assessors (Govaerts et al. 2007).

We do not presume that our model will produce the ‘expert assessor’ of the future who can overcome any conceivable assessment challenge, for, indisputably, even the most expert assessor is prone to biases (Plous 1993), and it takes a carefully developed program of assessment to tackle issues like the ‘content’ and ‘context specificity’ of performance (Turnbull et al. 1996; Norman et al. 1985). Nevertheless, their large store of ‘performance scripts’ allows expert assessors to use top-down information processing (Evans 2008) thereby freeing up cognitive resources (van Merrienboer and Sweller 2010) and alleviating feelings of uncertainty.

Such feelings in the face of complex and ill-defined problems are not unique to performance assessment. They are an equally familiar feature of clinicians’ experiences in dealing with complicated patient problems or choosing between different treatment options. Clinicians usually respond to doubt by running additional tests, and the participants in our study similarly called for supplementary assessment opportunities in cases of doubt. Knowing there will be further occasions where a student’s performance on a certain task will be scrutinized, it will be easier for assessors to acknowledge dilemmas over appropriate judgment of present performance. However, examination rules and procedures generally preclude this approach, causing assessors to give students a pass despite doubts about the adequacy of their performance.

Acknowledging the interdependence of quality of assessment and tolerance of uncertainty is even more important from the perspective that performance assessment in medical education is conducted in and affected by the social environment. This factor is acknowledged by our preliminary conceptual framework which is in line with research in the fields of medical education (Govaerts et al. 2007), social perception, and social cognition (Murphy and Cleveland 1995; Levy and Williams 2004).

In line with the aim of the study, the findings have implications for developing measures to improve the practice of performance assessment in undergraduate medical education. First, our model suggests principles to guide the design of faculty development activities aimed at enhancing assessors’ expertise in individual assessment tasks. Activities like one time briefings explaining the use of a specific assessment form are likely to have few, if any, lasting effects. More promise seems to be offered by a longitudinal trajectory incorporating elements of deliberate practice and peer feedback and which should be integrated in teachers’ daily assessment tasks.

Our findings highlight the desirability of assessment programs designed to counteract assessors’ insecurities due to dilemmas over student performance by affording additional assessment opportunities to build a more convincing basis for decisions. Such programs could create an environment where assessors feel free to openly communicate their doubts thereby ameliorating assessors’ anxiety caused by uncertainty in assessing borderline performance.

In summary, in this study we present a preliminary model of factors contributing to assessor expertise in single assessments of student performance. The findings are firmly grounded in empirical data and in line with literature on expertise development and with findings from social cognition research. As this was a study in a single institution, the transferability of the results may be limited, although we purposively sampled participants to represent different professional backgrounds, gender, and assessment experience, and all participants had assessment experience in two or more different medical programs. Future studies should examine the validity of the model at other locations. The implications for medical education practice also warrant further investigation.
